# Rethinking Molecular Mimicry in Rheumatic Heart Disease and Autoimmune Myocarditis: Laminin, Collagen IV, CAR, and B1AR as Initial Targets of Disease

**DOI:** 10.3389/fped.2014.00085

**Published:** 2014-08-19

**Authors:** Robert Root-Bernstein

**Affiliations:** ^1^Department of Physiology, Michigan State University, East Lansing, MI, USA

**Keywords:** antigenic complementarity, epitope drift, hidden antigens, theory, co-infection, group A streptococci, coxsackie virus, hyperinflammation

## Abstract

**Rationale:** Molecular mimicry theory (MMT) suggests that epitope mimicry between pathogens and human proteins can activate autoimmune disease. Group A streptococci (GAS) mimics human cardiac myosin in rheumatic heart disease (RHD) and coxsackie viruses (CX) mimic actin in autoimmune myocarditis (AM). But myosin and actin are immunologically inaccessible and unlikely initial targets. Extracellular cardiac proteins that mimic GAS and CX would be more likely.

**Objectives:** To determine whether extracellular cardiac proteins such as coxsackie and adenovirus receptor (CAR), beta 1 adrenergic receptor (B1AR), CD55/DAF, laminin, and collagen IV mimic GAS, CX, and/or cardiac myosin or actin.

**Methods:** BLAST 2.0 and LALIGN searches of the UniProt protein database were employed to identify potential molecular mimics. Quantitative enzyme-linked immunosorbent assay was used to measure antibody cross-reactivity.

**Measurements:** Similarities were considered to be significant if a sequence contained at least 5 identical amino acids in 10. Antibodies were considered to be cross-reactive if the binding constant had a *K*_d_ less than 10^-9^ M.

**Main results:** Group A streptococci mimics laminin, CAR, and myosin. CX mimics actin and collagen IV and B1AR. The similarity search results are mirrored by antibody cross-reactivities. Additionally, antibodies against laminin recognize antibodies against collagen IV; antibodies against actin recognize antibodies against myosin, and antibodies against GAS recognize antibodies against CX. Thus, there is both mimicry of extracellular proteins and antigenic complementarity between GAS-CX in RHD/AM.

**Conclusion:** Rheumatic heart disease/AM may be due to combined infections of GAS with CX localized at cardiomyocytes that may produce a synergistic, hyperinflammatory response that cross-reacts with laminin, collagen IV, CAR, and/or B1AR. Epitope drift shifts the immune response to myosin and actin after cardiomyocytes become damaged.

## Introduction

The purpose of this study is to re-evaluate the role of molecular mimicry (MM) in rheumatic heart disease (RHD) and autoimmune myocarditis (AM) in light of serious questions concerning its adequacy to explain autoimmune disease.

The basic idea behind molecular mimicry theory (MMT) is that microbes evolve to evade the immune system through selection for proteins that mimic those of their host (1–5). One result of this mimicry is cross-reactivity between pathogens and host proteins that can trigger autoimmune disease. Several pathogens have been associated clinically and epidemiologically with RHD and AM: group A streptococci (GAS) [reviewed in Ref. (6, 7)]; coxsackie viruses (CX) [reviewed in Ref. (8, 9)]; *Trypanosoma cruzi* (10); Chlamydia (11, 12), perhaps varicella zoster virus (13), cytomegalovirus (14), hepatitis C virus (15), ECHO virus, adenovirus, Epstein–Barr virus, and parvovirus B19 (16–19). It was established several decades ago that three of these pathogens, GAS, *T. cruzi*, and CX induce immunological responses that cross-react with cardiac proteins (20–26). The demonstration that GAS induced myosin-specific antibodies that were associated with organ-specific disease and that RHD could be induced using cardiac myosin or its mimic, the M protein of GAS, seemed to clinch the case for MM as the cause of autoimmune disease. Concomitant development of animal models of AM using CX infections (27, 28) further substantiated MMT.

Serious questions, however, have been raised over the past few years as to the adequacy of MMT to explain autoimmune diseases in general and RHD and AM in particular. If MM is sufficient to induce autoimmune disease, then one would expect much higher rates of disease than are observed. All of the infections associated with onset of RHD and AM are very common, yet few infections result in autoimmune disease. It is estimated that 14% of the population has genes associated with increased RHD risk (7, 29), most people contract one or more GAS or CX infections, yet only 1 in 5000 people who develop GAS pharyngitis go on to develop RHD (30, 31) while only about 1 in 500 people who develop a coxsackie viral infection develop AM (32). The rates of RHD and AM are thus far smaller than would be predicted based on MMT.

Another problem is that according to many investigators, very little evidence exists for MM playing a role in human disease (33–35). Fourneau et al. concede that the best human data exist for RHD and Guillain–Barre syndrome (GBS) but caution that even for RHD and GBS, it had not been demonstrated that the initial immune response to the target tissues in either disease is actually due to MM; it was quite possible that infection results in release of hidden antigens such as myosin and actin that add to, but do not induce, disease. Fujinami et al. has recently expressed reservations about the theory he himself helped to develop (36), noting that all current animal models based on MMT require adjuvants, in addition to the molecular mimic, to induce a hyperinflammatory response. It is not, for example, possible to induce RHD in animals using purified M proteins from GAS; experimental RHD requires the addition of Freund’s complete adjuvant – no other adjuvant has been found to work (32). The role of the adjuvant in MMT has remained unresolved but strongly suggests that mimicry is insufficient to induce autoimmune disease. Fujinami notes that molecular mimics may require bystander-induced damage to initiate disease by releasing hidden antigens (36), a position that has urged more strongly by Tandon et al. (37). The applicability of MMT to Chagas disease has also been hotly debated (38).

The major problem faced by MMT is that while molecular or epitope mimicry may be necessary, it is not clear that it is sufficient to explain autoimmune disease. In RHD/AM, most of the reported mimicry between pathogens and host proteins involve intracellular host proteins such as cardiac myosin (6, 26, 39) and actin (40). These proteins should not be accessible to the immune system as long as cardiac tissue is intact. Neu et al. (41) have, in fact, demonstrated that anti-myosin antibodies do not recognize intact myocardiocytes, that the myosin antibody titer does not correlate with onset of experimental autoimmune myocarditis (EAM), and that myosin antibodies are not sufficient to induce EAM in a passive transfer model. In addition, as Reddy et al. (42) has argued, if myosin were the major autoantigen inducing RHD and AM, then one would expect that patients with cardiac damage would develop these diseases at much higher rates than other patients, yet it has been demonstrated that patients who contract rheumatic fever or have cardiac infarcts, bypass surgeries, and heart transplants do not develop RHD or AM despite producing anti-myosin antibodies (43–46). This problem is exacerbated by evidence that GAS and CX induce antibodies against many non-cardiac proteins and that non-pathogens mimic myosin as well as do GAS and CX (47–52). This produces the conundrum that the existence of mimicry is not sufficient to induce disease while non-pathogens, under the right conditions, can! In fact, in EAM, there is no immune response to cardiac myosin initially; anti-myosin antibodies develop later in the disease process (41, 53–55). An autoimmune response to some cardiac protein other than myosin must come first, so there must be some way of targeting heart tissue other than GAS-myosin mimicry. Why, then, are myosin antibodies so well correlated with RHD and AM pathogenesis and how does the immune system “know” to target tissues that contain antigens that it cannot “see” before it has damaged that tissue? Has the focus on GAS mimicry of myosin blinded us to other forms of epitope mimicry against cell surface targets such as the beta 1 adrenergic receptor (B1AR) that has been identified as a possible autoimmune target in dilated cardiomyopathies (DCM) (56–59)?

Despite the many problems that clearly challenge MMT, it would be a mistake to “throw the baby out with the bath water,” as the saying goes. Science does not progress by discarding the knowledge acquired by inadequate theories but by incorporating that knowledge into a more comprehensive theory. Each of the problems just enumerated helps to define the kinds of factors that need to be added to MMT to produce a more complete and effective theory of autoimmune disease. Davies (33) and Fourneau et al. (35) pointed out that antigens such as myosin and actin that dominate the late immune response in RHD/AM need not be the antigens that initiate autoimmunity; epitope spreading may occur in which one host protein is the initial target of an autoimmune response, the result of which is release of hidden host antigens that then come to dominate subsequent autoimmunity. Clues exist as to what proteins might be the initial targets in RHD/AM. GAS utilize collagen type IV in the basement membranes of cardiac tissue as binding sites (37, 60–62). GAS are also known to target specific tissues by means of cell surface proteins Cnm and Cbm that bind specifically to collagen type 1 and Cbm subtypes are found preferentially in heart biopsies from patients with endocarditis (63). In addition, GAS can use a hemoprotein receptor, (Shr), as well as an extracellular protein factor (Epf or PrtF), to bind to laminin and fibronectin on target cells (64–66). Similarly, type B CX and sub-group type C adenoviruses use the coxsackie and adenovirus receptor (CAR) and the CD55 or decay-accelerating factor for complement (DAF) to target cells [reviewed in Ref. (67)] but other extracellular proteins are clearly involved since antibodies against these receptors, and soluble receptors themselves, are not sufficient to prevent CX infection of cardiomyocytes (68, 69). Another possible target might be the beta 1 adrenergic receptor (B1AR), which has already been implicated in DCM (56–59). In short, one might expect that extracellular proteins such as laminins, collagens, fibronectin, DAF, CAR, and B1AR would be much more likely than intracellular proteins, such as myosin, actin, or troponin, to be initial targets of an autoimmune response producing cardiomyocyte damage.

The purpose of this paper is to explore the possibility that extracellular cardiac proteins employed by GAS and CX as receptors may be the initial targets of the autoimmune response in RHD/AM and that cardiac myosin and actin are actually secondary targets that help sustain the autoimmune response. More particularly, we investigate whether GAS and CX may mimic extracellular cardiac proteins and whether epitope drift may then shift the autoimmune response from external to internal protein targets. Two types of methods are employed. The first involves similarity searches to determine whether extracellular cardiac proteins mimic GAS and CX. The second involves the use of quantitative enzyme-linked immunosorbent assay (ELISA) to determine whether there is significant cross-reactivity between antibodies against GAS or CX and cardiac proteins.

## Materials and Methods

### Similarity searches

Two alignment methods, BLAST (70) and LALIGN (71), were employed to search for similarities between microbial and cardiac proteins. Both methods are limited to searching for linear epitope similarities, which will miss three-dimensional conformational similarities and possible cross-reactivities between protein and non-protein epitopes. Since most (though certainly not all) MM is likely to involve T-cell mediation, and T cells generally recognize linear peptides 8–20 amino acids in length, these limitations seem acceptable for the present study. Moreover, since both programs were designed to reveal evolutionary relationships rather than immunological ones, an additional criterion was imposed on all of the results, which was that any similarity had to involve at least 5 identical amino acids in a sequence of 10 and preferably additional identities or reasonable amino acid substitutions. Previous experiments have demonstrated that this 5-of-10 rule has good predictive value for identifying cross-reactive peptide epitopes (72, 73).

BLAST 2.0 (www.expasy.org) was used to explore the overall degree of similarity between the microbes and proteins shown in Tables 1 and 2. The BLAST parameters were set on the maximum values for the *E* threshold (10000), number of best scoring sequences to show (3000), and number of best alignments to show (1000); gapped similarities were permitted and the low complexity filter was turned off. Data on the number of similarities having 6 or more identities in a sequence of 10 amino acids and the number of sequences that had *E* values (a function measuring the probability of any two sequences in the protein database of equal length having an equivalently good match) greater than 60 were tabulated (Tables 1 and 2). The choice of these identity and *E* values clearly distinguished between proteins that had high degrees of similarity and those that did not.

**Table 1:** **Summary of results of BLAST 2.0 similarity searches comparing the human proteins (left) with the pathogens (top)**.

	CX 6	CX > 60	MP1 6	MP1 > 60	MP2 6	MP2 > 60	HCV 6	HCV > 60	PV6	PV > 60
Actin	3	3	3	0	1	0	2	0	6	0
Collagen I	8	0	1	1	1	1	15	6	5	1
Collagen IV	6	1	2	0	2	0	18	13	6	1
Troponin	5	0	5	1	9	1	2	0	2	1
Myosin	14	2	29	50	35	50	7	2	7	4
Laminin 1	8	4	3	4	4	3	1	0	9	2
Laminin 2	10	6	4	17	5	12	2	6	6	5
Laminin 3	4	3	5	11	7	11	4	3	8	3
Laminin 4	4	0	6	2	4	3	4	3	10	0
Laminin 5	10	2	7	8	5	8	2	5	3	1
CD55	5	0	0	0	0	0	7	0	6	1
CXAR	3	1	1	0	1	0	5	0	1	0
PVR	4	1	0	0	0	0	6	1	6	1
CD81	1	0	0	0	0	0	3	0	2	0
CLDN1	2	0	0	0	0	0	3	0	1	0
CD20	2	0	5	0	3	0	6	0	2	0
B1AR	1	1	0	0	0	0				
HepC	*	*	3	0	3	0				
PV	*	*	0	0	0	0				

*Actin is cardiac actin; Myosin is cardiac myosin; CD55 is also known as DAF, the complement decay-accelerating factor used by coxsackie viruses as a receptor; CXAR is the coxsackie and adenovirus receptor; PVR is the poliovirus receptor; CD81, a hepatitis C virus receptor; CLDN1 is claudin 1, a cell adhesion molecule; CD20, a B lymphocyte antigen; B1AR is the beta 1 adrenergic receptor, an autoantibody target in cardiomyopathies. CX is coxsackie B3 virus; MP1 and MP2 are two versions of the group A streptococcal M protein; HCV is hepatitis C virus; PV is poliovirus. In the columns labeled CX, etc. followed by “6” are listed the number of similar sequences that contained at least 6 identical amino acids in a sequence of 10. In the columns labeled CX, etc. followed by “ > 60” are listed the number of similar sequences that had an *E* value of greater than 60. *E* values greater than 60 are generally rare and usually indicate lengthy and statistically significant regions of similarity. The most significant sets of similarities as determined by a combination of both identical amino acids and *E* values are highlighted in shades of gray (significant), dark gray (very significant), or black (extremely significant) as determined by comparison with the rest of the study results*.

**Table 2:** **Summary of BLAST 2.0 similarity searches comparing human proteins (left) with each other (top)**.

	Myo 6	Myo > 60	Act 6	Act > 60	Col1 6	Col1 > 60	LS-4 6	LS-4 > 60	Tro 6	Tro > 60
Actin	2	0			0	0	0	0	1	0
Collagen I	2	0	0	0			10	1	0	0
Collagen IV	3	0	2	1	*	*	12	2	1	0
Troponin	6	3	1	0	0	0	3	1		
Myosin			2	0	2	0	3	4	6	3
Laminin 1	6	12	0	0	10	2	*	*	2	1
Laminin 2	4	22	4	0	24	20	*	*	6	0
Laminin 3	7	21	3	0	16	4	*	*	2	0
Laminin 4	22	10	0	0	9	1			3	1
Laminin 5	5	16	7	0	12	10	*	*	9	1
CD55	3	0	0	0	3	0	1	0	0	0
CXAR	2	0	0	0	0	1	4	0	0	0
PVR	0	0	0	0	1	0	0	0	0	0
CD81	1	0	0	0	0	0	0	1	1	0
CLDN1	0	0	0	0	3	0	2	0	1	0
CD20	5	0	0	0	2	0	2	0	1	0

*Myo stands for cardiac myosin; Act for actin; LS-4 for laminin type 4; and Tro for troponin. The meanings of the “6” and “ > 60” are the same as in Table 1, as are the highlights*.

**Figure 1:**
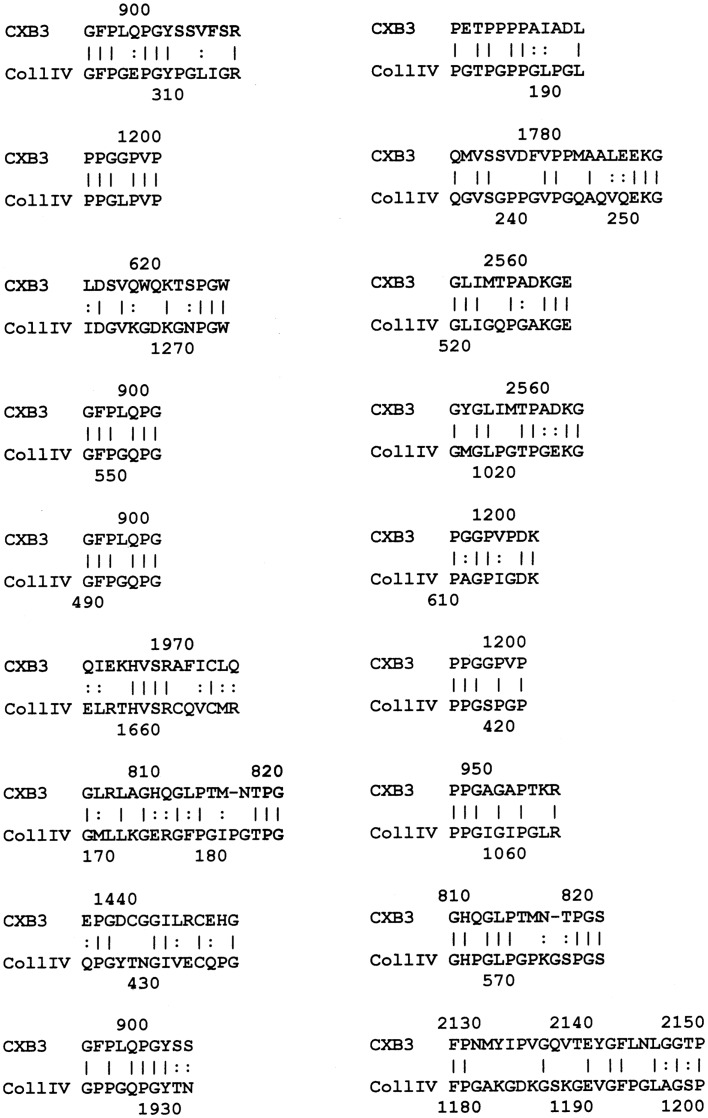
**Results of LALIGN similarity search comparing coxsackie B3 virus (P03313) to human collagen IV (P02462)**.

**Figure 2:**
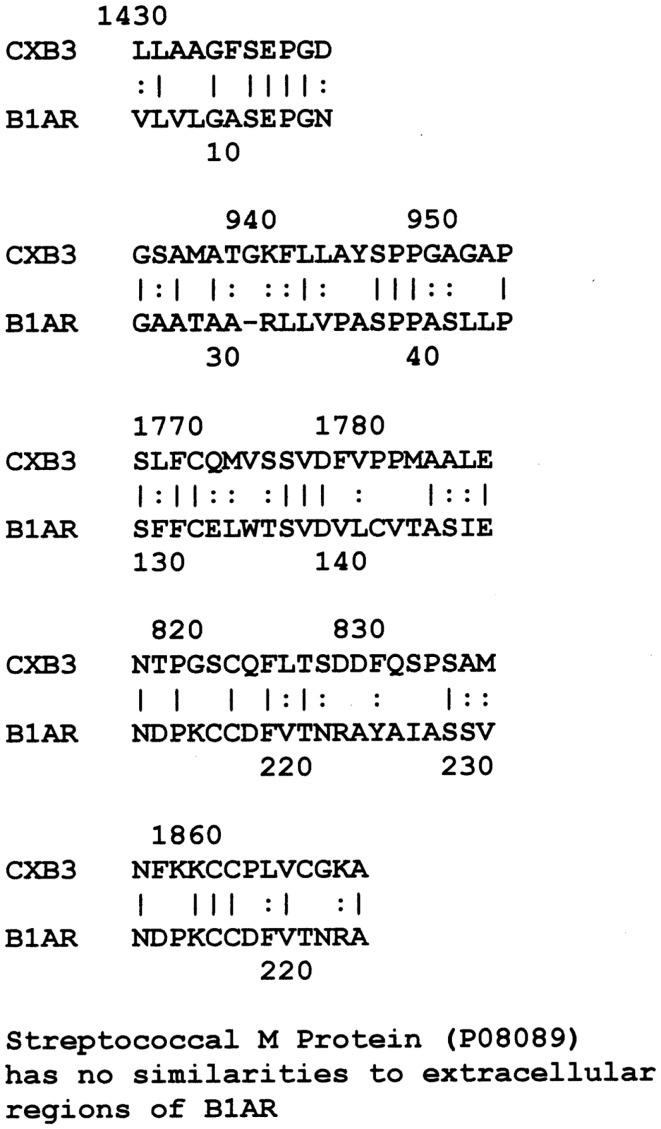
**Results of LALIGN similarity search comparing coxsackie B3 virus (P03313) to the extracellular regions of the cardiac human beta 1 adrenergic receptor (B1AR) (P08588)**. GAS M proteins, notably, have no sequence similarity with B1AR.

**Figure 3:**
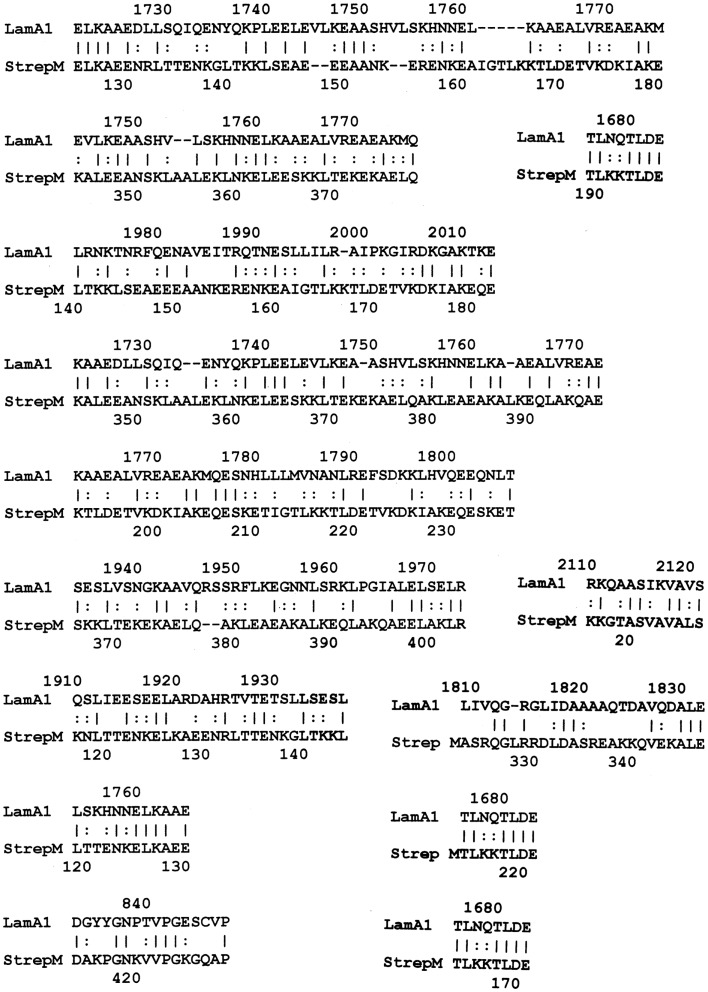
**Results of LALIGN similarity search comparing group A streptococcal M protein (P08089) to human laminin alpha 1 (P25391)**.

**Figure 4:**
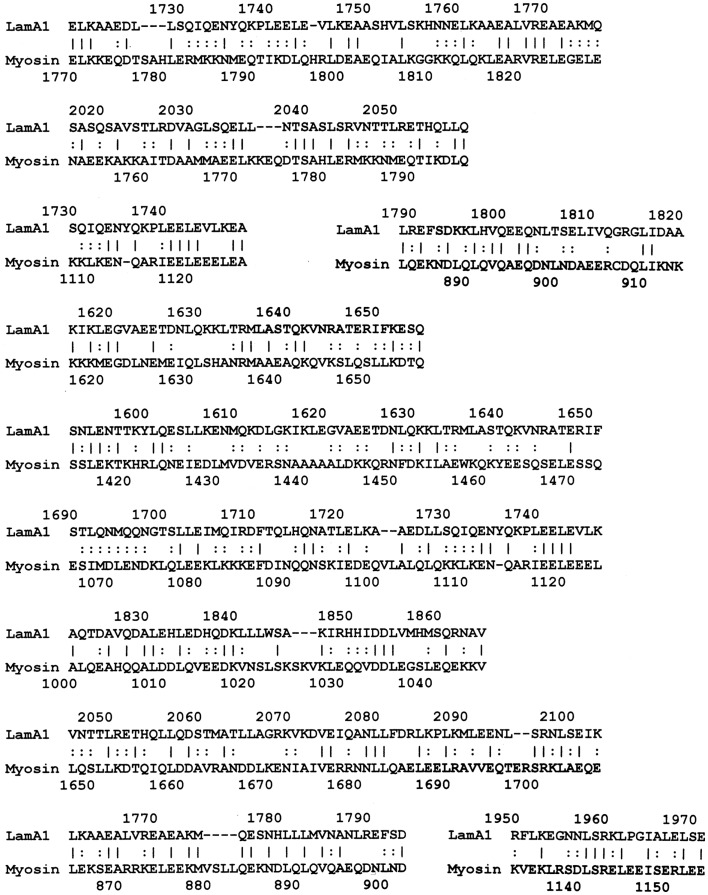
**Results of LALIGN similarity search comparing human laminin alpha 1 (P25391) to human cardiac myosin (P13533)**.

**Figure 5:**
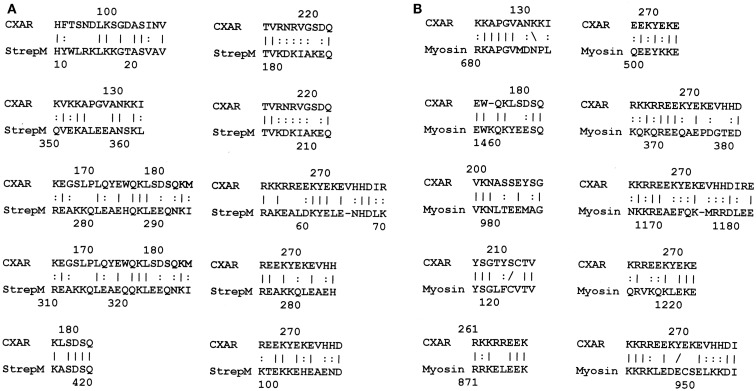
**(A)** Results of LALIGN similarity search comparing human coxsackie and adenovirus receptor (CAR) (P78310) to group A streptococcal M protein (P02977); and **(B)** results of LALIGN similarity search comparing human coxsackie and adenovirus receptor (CAR) (P78310) to human cardiac myosin (P13533).

The results of the BLAST search was refined using LALIGN (www.expasy.ch), which permits better targeted similarity searches, and these results were used to investigate the specific regions of the proteins involved in potential epitope similarities. The LALIGN parameters were set on the maximum number of alignments (20) using BLOSSUM80 leaving all other settings were in default mode. The results of the LALIGN searches are shown in Figures 1–5.

### Experiments

Three types of experiments, ELISA, double-antibody ELISA (DA-ELISA), and Ouchterlony immunodiffision, were employed to investigate whether the similarity searches yielded immunologically valuable information.

Enzyme-linked immunosorbent assay (ELISA) was used to investigate cross-reactivities between microbial antibodies and cardiac tissue-related proteins. The tissue protein was diluted in pH 7.4 phosphate buffer to a concentration of 10 μM. This standard solution was then diluted by 10-fold steps to about 10^−14^ M. Two wells received only phosphate buffer as controls. 100 μL of each protein dilution was added in duplicate to wells of a Costar round-bottomed 96-well ELISA plate and incubated for 1 h. The excess protein triply washed out using a 1% Tween 20 solution (in phosphate buffer) and a plate washer. Next, 200 μL of blocking agent (2% polyvinylalcohol in phosphate buffer) was added to every well, incubated for an hour, and then triply washed. An antibody against a microbe (at 1 mg/mL concentration) was then diluted to 1/200 in phosphate buffer and 100 μL added to every well. The antibody was incubated for an hour and then triply washed. A species-appropriate horseradish peroxidase-linked secondary antibody was then at a dilution of 1/1000, incubated for an hour, and triply washed. Finally, 100 μL of ABTS reagent (Chemicon) was added, incubated for 30 min, and the plate read at 405 nm in a Spectramax UV-VIS scanning spectrophotometer. Data were gathered using Spectramax software and then analyzed using Excel. Analysis essentially consisted of subtracting non-specific binding to the buffer-only wells from the protein-containing wells and plotting the amount of antibody binding (as measured by absorbance at 405 nm) as a function of protein concentration.

Double-antibody ELISA was used to investigate possible antigenic complementarity between the antibodies used in the study. DA-ELISA differs from ELIAS in that the protein laid down in n the 96-well plate in the initial step of an ELISA is substituted with an antibody. A second antibody (from a different species) is tested for its ability to bind to the first. The ability of the second antibody to bind to the first is then monitored using peroxidase-linked antibody against the species from which the second antibody is derived (74–77). As in the ELISA protocol, the first antibody is made up at a concentration of about 10 μM (assuming IgG antibodies have a molecular weight of 180,000 daltons) and then serially diluted by factors of 10. The rest of the protocol is the same.

Finally, since DA-ELISA is limited to testing whether antibodies from different species interact, Ouchterlony immunodiffusion was used to determine whether pairs of monoclonal antibodies might recognize each other as antigens (75, 76). A 2.5% agarose gel was made using distilled, deionized water; the gel was plated on sterile microscope slides; when the gel had set, holes were punched in the gel using a template; and 30 μL of each monoclonal antibody was added to each well. The slides were checked for precipitation lines after 24 h.

### Antibodies

#### Group A Streptococcal antibodies

Pierce GAS monoclonal antibody MA1-10698; Pierce GAS monoclonal antibody MA1-10699; Pierce GAS monoclonal antibody MA1-10700; Pierce GAS monoclonal antibody MA1-10701; MyBioSource Group A Streptococcus monoclonal antibody MBS190189; Rabbit anti-Streptococcus group A-HRP antibody (AcrisBP2026HRP).

#### Coxsackie antibodies and antisera

Millipore Ms x Coxsackie Virus B3 MAB948; USBiological Ms x Coxsackie Virus B3 MAb C7904-91E; USBiological Ms x CX B1-6; Biorbyt Ms x Coxsackie Virus B3 orb79434; Horse anti-coxsackie B3 virus antiserum (NIH ATCC); Monkey anti-coxsackie B4 virus antiserum (NIH ATCC).

#### Protein antibodies

Rabbit anti-actin antibody (Sigma A2668); Rabbit anti-laminin antibody (Sigma L9393); Goat anti-cardiac myosin (Santa Cruz Biotechnology sc-12117); Rabbit anti-collagen IV (Novus Biologicals NB120-6586).

#### Secondary antibodies

Goat anti-mouse IgG-HRP antibody (Sigma A9917); Goat anti-rabbit-HRP (Sigma A0545); Goat anti-human IgG-HRP (Chemicon AP120P); Goat anti-horse HRP (Biodesign W99260P).

#### Proteins

Myosin, calcium activated from porcine heart (Sigma-Aldrich MO531); Actin from rabbit muscle (Sigma-Aldrich A2522); Human Fibronectin (Cultrex); Human Collagen I (Cultrex); Human Collagen IV (Cultrex); Mouse Laminin 1 (Cultrex); Human Vitronectin (Cultrex).

## Results

Significant molecular and antigenic mimicry between myosin and the M protein of GAS (1, 30, 31) and between cardiac actin and CX (40) has been reported previously and the BLAST and LALIGN similarity searches strongly confirm these previous reports (Table 1). One question addressed in this research is whether GAS and CX have significant similarities to other cardiac proteins, particular extracellular ones, that might explain the cardiotropic effects of GAS- and CX-related autoimmune disease. Such similarities do exist using two measure of similarity, number of sequences that share 6 or more identical amino acids out of 10, and the number of shared sequences that have *E* scores of 60 or above (indicating statistically significant sequence similarity over extended stretches). A BLAST 2.0 search of GAS and CX proteins against various human cardiac proteins yielded the results summarized in Tables 1 and 2. Table 1 shows that CX mimics cardiac actin, collagens, myosin, and some laminins but not troponin or a variety of receptors such as the CAR, the poliovirus receptor, etc. The M protein of GAS shares, as expected from previous studies, many significant sequences with cardiac myosin, and also with laminins, but with no other proteins studied. Table 2 demonstrates that the GAS similarities to myosin and laminins are reflected in very significant similarities between myosin and laminins themselves. Myosin also shares some sequence similarity to troponin. Collagens I and IV share significant sequence similarities with some laminins as well. BLAST similarity searches therefore suggest that MM extends beyond myosin-GAS or actin-CX to include extracellular matrix proteins.

LALIGN similarity searches, which are more targeted than BLAST searches, identified many individual regions of similarity between the various proteins tested in this study. Significant similarities were defined by the presence of at least 5 identical amino acids in a sequence of 10, and counting every pair of acceptable amino acid substitutions as an identity. While more than a dozen significant similarities were found between actin and CX proteins, these are not illustrated here as some of them have been published previously (40). Figure 1 shows that CXB3 also has many significant similarities to collagen IV. It follows both logically and evidentially, that cardiac actin and collagen IV also share many significant similarities and these, too, were observed (data not shown). Thus, CX, actin, and collagen IV share potential epitope mimicry among themselves. CX also mimics several of the extracellular sequences of the B1AR (Figure 2), which has been identified as a target of autoantibodies in dilated cardiomyopathy (56–59).

LALIGN studies revealed a similar set of potential epitope mimics among GAS, myosin, laminin, and the CAR. Cunningham (1, 30, 31) has already established the very significant sequence similarities and cross-reactivities between GAS M protein and cardiac myosin, so data pertinent to that fact have been omitted here. Surprisingly, laminins also share very significant sequence similarities to GAS M protein (Figure 3 illustrates the data for laminin alpha 1 only, but other laminins share similarly significant regions). In addition, laminins share very significant similarities with human cardiac myosin (again illustrated only with laminin alpha 1 data in Figure 4). Even more surprisingly, CAR shares potential epitope similarities with laminins (data not shown), GAS M protein (Figure 5A), and cardiac myosin (Figure 5B). Thus, it is possible that CAR is a target of the anti-GAS, anti-myosin, and anti-laminin antibodies that are known to accompany RHD/AM.

ELISA was used to test whether the BLAST and LALIGN results translate from sequential similarities to actual antibody cross-reactivities. Table 3 summarizes the results of the studies carried out here, which investigated cross-reactivities between anti-GAS and anti-CX antibodies and extracellular matric proteins, but not the beta 1 adrenergic receptor or CAR, which were not readily available in the quantities necessary for such experiments. In general, antibodies raised against a protein target yield dissociation constants in the 10^−12^ M range in these studies, whereas dissociation constants due to cross-reactivity between antibodies raised against one protein that recognize another may range between 10^−11^ and 10^−6^ M. Those cross-reactivities that have dissociation constants less than 10^−9^ have been highlighted in Tables 3 and 4 as having the greatest probability of being of pathological significance.

**Table 3:** **Results of ELISA experiments involving GAS (group A streptococcus) and CX (coxsackie virus) antibodies binding to the proteins listed along the top**.

	Laminin	Myosin	Actin	Collagen IV	Collagen I	Fibronectin	Vitronectin
GAS MA1-10698 (62)	**2 ***×*** 10^***−***9^**	**2 ***×*** 10^***−***10^**	2 × 10**^−^**^6^	1 × 10**^−^**^7^	**4 ***×*** 10^***−***9^**	1 × 10**^−^**^6^	3 × 10**^−^**^8^
GAS MA1-10699 (64)	>10**^−^**^5^	>10**^−^**^5^	>10**^−^**^5^	>10**^−^**^5^	>10**^−^**^5^	>10**^−^**^5^	>10**^−^**^5^
GAS MA1-10700 (65)	8 × 10**^−^**^6^	**3 ***×*** 10^***−***10^**	>10**^−^**^5^	3 × 10**^−^**^7^	>10**^−^**^5^	1 × 10**^−^**^7^	>10**^−^**^5^
GAS MA1-10701 (66)	>10**^−^**^5^	>10**^−^**^5^	>10**^−^**^5^	>10**^−^**^5^	>10**^−^**^5^	>10**^−^**^5^	>10**^−^**^5^
GAS Mab MBS190189	**4 ***×*** 10^***−***9^**	3 × 10**^−^**^8^	>10**^−^**^5^	>10**^−^**^5^	1 × 10**^−^**^7^	4 × 10**^−^**^8^	>10**^−^**^5^
GAS Rabbit-HRP	>10**^−^**^5^	>10**^−^**^5^	>10**^−^**^5^	>10**^−^**^5^	>10**^−^**^5^	>10**^−^**^5^	>10**^−^**^5^
CXB4 Horse	3 × 10**^−^**^7^	3 × 10**^−^**^8^	**2 ***×*** 10^***−***9^**	**1 ***×*** 10^***−***11^**	1 × 10**^−^**^6^	>10**^−^**^5^	>10**^−^**^5^
CXB3 Monkey	>10**^−^**^5^	3 × 10**^−^**^8^	**7 ***×*** 10^***−***10^**	**2 ***×*** 10^***−***9^**	>10**^−^**^5^	**8 ***×*** 10^***−***10^**	>10**^−^**^5^
CXB3 MAB948	1 × 10**^−^**^7^	5 × 10**^−^**^8^	>10**^−^**^5^	>10**^−^**^5^	>10**^−^**^5^	1 × 10**^−^**^6^	>10**^−^**^5^
Anti-laminin Rabbit	**7 ***×*** 10^***−***12^**						

*The numbers represent the binding constants of the antibodies to the proteins using the inflection point of the binding curves as an approximation of the binding constants (see Figures 10 and 11). The smaller the binding constant, the greater the affinity of the antibody for the protein. Rabbit anti-laminin binding to laminin is provided as a reference for the binding expected of an antibody to the target against which it was raised*.

**Table 4:** **Results of double-antibody (DA)-ELISA experiments and Ouchterlony immunodiffusion experiments involving GAS (group A streptococcus) antibodies binding to CX (coxsackie virus) antibodies**.

	HS CXB4	MN CXB3	CXB1-6 MA	CBXB3 MAB1	CXB3 MAB2	CXB3 MAB3
GAS MA1-10698 (62)	**4 ***×*** 10^***−***11^**	**5 ***×*** 10^***−***9^**	0	0	0	0
GAS MA1-10699 (64)	**6 ***×*** 10^***−***11^**	**6 ***×*** 10^***−***9^**	0	0	0	0
GAS MA1-10700 (65)	**8 ***×*** 10^***−***11^**	**4 ***×*** 10^***−***9^**	0	0	0	0
GAS MA1-10701 (66)	**3 ***×*** 10^***−***11^**	**5 ***×*** 10^***−***9^**	0	0	0	0
GAS Mab MBS190189	**5 ***×*** 10^***−***10^**	1 × 10^−7^	0	0	0	0
GAS Rabbit-HRP	>10^−5^	>10^−5^	0	0	0	0
Goat anti-Horse-HRP	**1 ***×*** 10^***−***12^**					

*Numbers represent the approximate binding constants of the antibodies for each other assuming a molecular weight of 180 kD for IgG antibodies and an average of one-to-one binding between antibodies. No pair of monoclonal antibodies (MA or MAB) precipitated each other in the Ouchterlony immunodiffusion experiments. Goat anti-Horse–Horseradish peroxidase-linked antibody binding to horse antibodies is provided as a reference for the binding affinity expected of an antibody to the target against which it was raised*.

**Figure 6:**
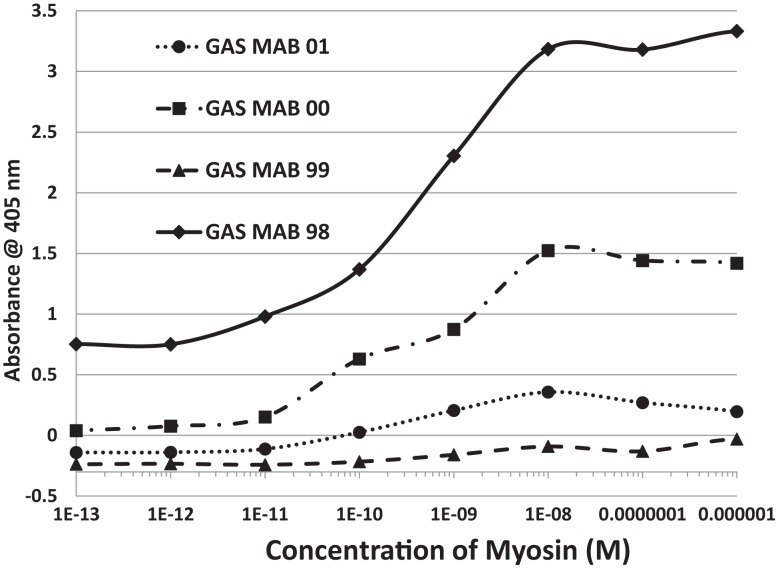
**Results of ELISA experiments involving monoclonal antibodies against group A streptococci (GAS MAB) binding to myosin**. Binding constants for Table 3 were derived from the inflection points of the curves.

**Figure 7:**
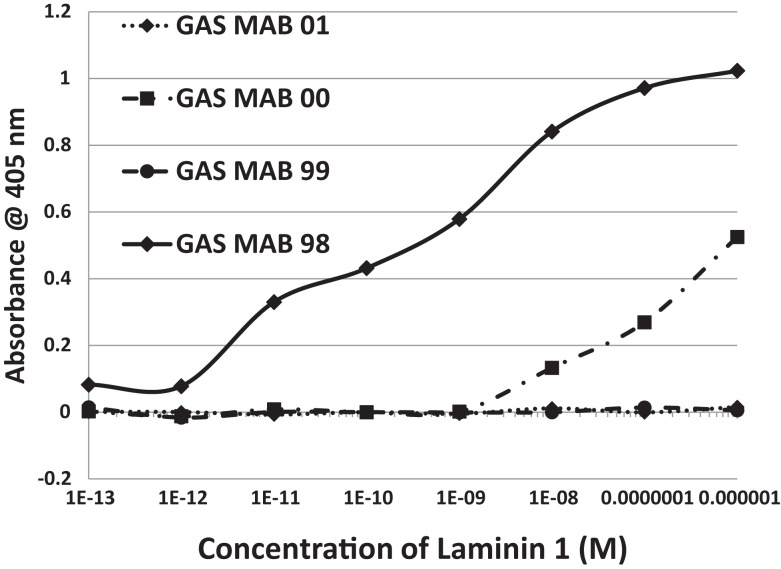
**Results of ELISA experiments involving monoclonal antibodies against group A streptococci (GAS MAB) binding to laminin 1**. Binding constants for Table 3 were derived from the inflection points of the curves.

In summary, while several antibodies against GAS had little or no significant cross-reactivity to any of the proteins tested, three monoclonal antibodies against GAS were identified that bound with significant affinity to both myosin and laminin. These antibodies generally had much less affinity for actin, collagens, fibronectin, and vitronectin. Some of the experimental data from these experiments are illustrated in Figures 6 and 7. These data suggest that GAS can induce antibodies that cross-react with laminins and that some of these antibodies recognize both laminins and myosin. These data are in accord with the BLAST and LALIGN results reported above.

**Figure 8:**
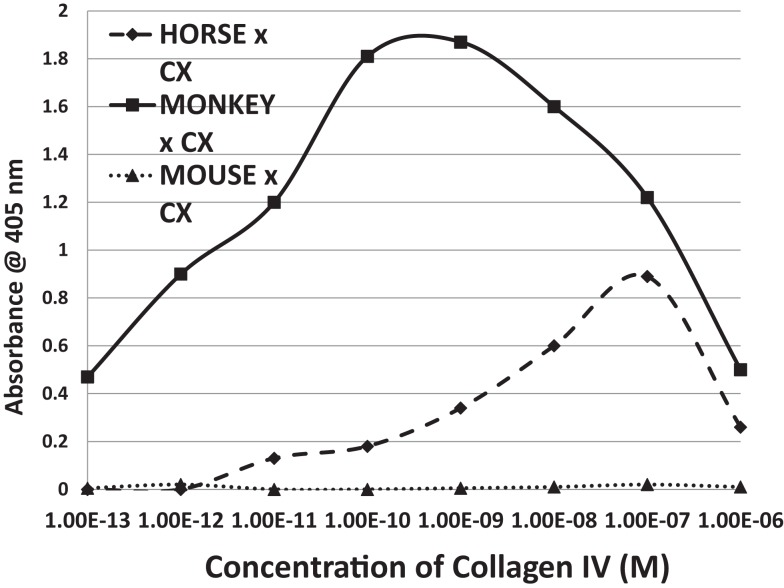
**Results of ELISA experiments involving horse anti-coxsackie B4 serum, monkey anti-coxsackie B3 serum, or a monoclonal (MAB) antibody against coxsackie B3 binding to human collagen IV**. The downward turn of the curves at high concentrations of collagen IV suggest that collagen IV self-aggregates, hiding the binding sits from antibody. Binding constants for Table 3 were derived from the inflection points of the curves.

Antibodies against CX raised in horses and monkeys, in contrast to those against GAS, had less affinity for laminin and myosin and more for actin, collagen IV, and fibronectin. Since CX antibody binding to actin has been documented elsewhere (40), those data are not illustrated here. A monoclonal antibody against CXB3 had primary affinity for myosin and little affinity for any of the other proteins tested. The binding of these CX antibodies to collagen IV is illustrated in Figure 8. These data suggest that CX can induce antibodies that cross-react with actin and collagen IV and, potentially, with fibronectin as well. Again, these results fit well with the predictions made from BLAST and LALIGN above. These data are summarized in Table 3.

**Figure 9:**
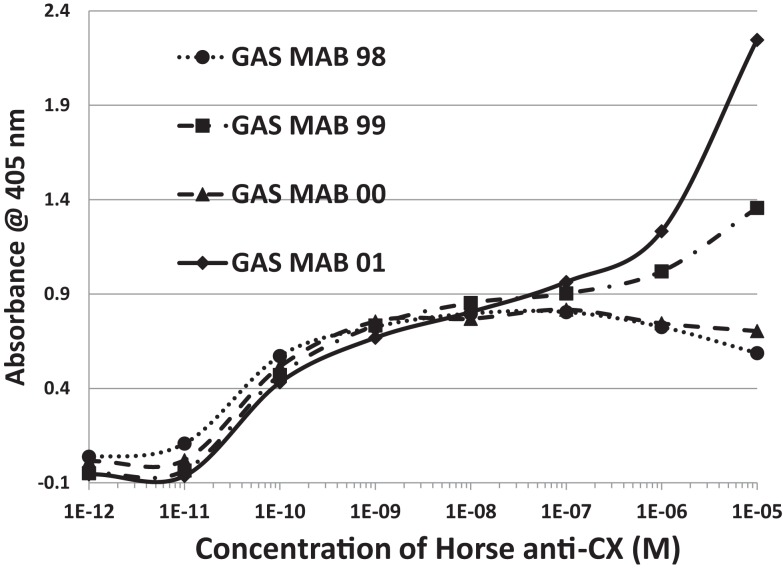
**Results of double-antibody ELISA (DA-ELISA) experiments involving monoclonal antibodies against group A streptococci (GAS MAB) binding to horse anti-coxsackie B4 serum**. Binding constants for Table 4 were derived from the inflection points of the curves. Note that two of the antibodies also display a second, low affinity binding to each other at high concentrations of CX antiserum. Very similar results were obtained for group A streptococci (GAS MAB) binding to monkey anti-coxsackie B3 serum (data not shown).

Several lines of reasoning involving molecular complementarity led to a further set of experiments. Both actin and myosin are targets of autoantibodies in RHD/AM and actin and myosin bind to each other through molecular complementarity. Both laminins and collagen IV are targets of autoantibodies in RHD/AM and laminin binds to collagen IV through molecular complementarity. GAS mimics laminins and myosin, while CX mimics actin and collagen IV, suggesting that GAS may have antigenic epitopes complementary to those of CX. In order to test whether such antigenic complementarities exist, DA-ELISAs were run in which the antigen in a typical ELISA is replaced with an antibody. Figure 9 shows that four of the monoclonal antibodies against GAS used in these studies bind with significant affinity to Horse x CXB4 antibodies, with a *K*_d_ of about 10^−10^ to 10^−11^. The same GAS antibodies bound to monkey CXB3 with *K*_d_’s of about 10^−8^ to 10^−9^ (data not shown). Several monoclonal antibodies against CX antigens were explored for binding to the GAS monoclonal antibodies using Ouchterlony immunodiffusion, but without success (Table 4), demonstrating that the GAS-CX complementarity involves specific, but as yet unidentified, antigens.

We have previously demonstrated similar binding between polyclonal GAS antibodies and the CX antibodies utilized here as well as anti-myosin antibody binding to CX antibodies (40). Since the BLAST and LALIGN similarity searches demonstrated significant mimicry between GAS M protein and laminin, and since laminin is very similar to myosin as well, GAS antibodies were replaced with anti-laminin antibodies in the next set of experiments. Anti-laminin antibodies also bind to CX antibodies with reasonably high affinity (*K*_d_’s of approximately 10^−9^ to 10^−10^ M) (data not shown). These data therefore demonstrate that CX antigens are complementary to GAS, myosin, and laminin antigens. This interpretation of the data is strengthened by the fact that laminin antibodies precipitate collagen IV antibodies in Ouchterlony immunodiffusion experiments (data not shown), as would be expected since collagen IV mimics some CX antigens (Figure 1, LALIGN data above).

The antibody specificities and antibody–antibody interactions were subject to a wide range of control experiments, the results of which are summarized in Tables 3 and 4. These controls demonstrate that the sets of cross-reactions and interactions are quite specific, the majority of control experiments yielding no measurable binding of the antibodies to proteins that are not major targets in RHD/AM.

To summarize, BLAST 2.0 and LALIGN search results demonstrate very significant similarities between the M protein of GAS, cardiac myosin, and laminins and also between CX proteins, cardiac actin, and collagen IV but not between GAS or CX and most other extracellular proteins or between myosin or actin and other extracellular proteins. Laminin also mimics the CAR, which mimics M protein and myosin as well. The cross-reactivities displayed *in vitro* by antibodies to GAS, CX, myosin, actin, collagen IV, and laminin are consistent with the BLAST results. Anti-GAS MAbs that recognize myosin also recognized laminin. Anti-CX antibodies have, instead, greater specificity for both actin and collagen IV. In other words, the M protein of GAS mimics both extracellular laminin and intracellular myosin, while CX proteins mimic both extracellular collagen IV and intracellular actin. Possible cross-reactivity between antibodies to CX and the B1AR and between antibodies to GAS and CAR are also possible.

## Discussion

### Placing the results in the context of the clinical and experimental literature

The *in vitro* findings described in the Section “Results” broaden the set of host antigens that are likely targets in RHD and AM beyond myosin and actin to include extracellular proteins associated with GAS and CX binding. These *in vitro* results are consistent with *in vivo* experimental and human clinical observations that have found autoantibodies against laminin, collagen IV (but not collagen I), B1AR, and CXAR as well as against myosin and actin in RHD and AM (39, 60–62, 78–85). In some cases, these antibodies recognize both laminin and cardiac myosin, mimicking the cross-reactivity observed *in vitro* here (81).

These clinical results support the extrapolation of the present results to understanding RHD/AM pathogenesis and permit some novel predictions to be made. Since extracellular matrix proteins are also molecular mimics of myosin, actin, GAS, and CX proteins, MM is much more widespread than previously thought. Specifically, laminin mimics GAS M protein and is recognized by some GAS antibodies; collagen IV mimics some CX proteins and is recognized by CX antibodies. In addition, laminin mimics cardiac myosin and laminin antibody recognizes myosin; collagen IV mimics cardiac actin and actin antibody recognizes collagen IV as an antigen. Among the possibilities that these results raise is that laminin is one of the receptor proteins used by CX to target cardiac tissues. Such a possibility is consistent with Orthopoulos et al.’s (68, 69) results showing that at least five, as yet unidentified, extracellular proteins besides CAR and DAF/CD55 are utilized by CX as receptors. Similar reasoning leads to the suggestion that GAS may utilize the beta 1 adrenergic receptor (B1AR) as a receptor, since B1AR mimics the already-identified GAS receptor collagen IV.

Recognition that molecular (or epitope) mimicry is more widespread in RHD/AM helps to resolve one problem – how intracellular proteins such as myosin become targets in RHD/AM – but exacerbates another, which is that laminins, collagens, and even DAF and CAR are fairly widespread proteins that are certainly not limited to cardiac tissue. So how do infections with GAS or CX result specifically in RHD or AM rather than some other autoimmune disease?

While genetic predisposition is probably one reason that some people are more susceptible to RHD/AM than others [e.g., (7, 29)], animal models of these diseases provide additional clues as to what produces autoimmune disease susceptibility. In all existing animal models of RHD and AM, even in susceptible strains of animals, the “causative” agents must always be accompanied by appropriate “adjuvants.” The M protein of GAS requires FCA (86); allogeneic myosin requires FCA (53, 87); CX requires either FCA boosted with pertussis or CX inoculated with cardiac alloantigens (28, 88, 89). CX passaged on tissues other than heart tissue does not produce EAM. Where FCA is required, no other adjuvant can be substituted (86). Use of incomplete Freund’s adjuvant (IFA) with myosin or M protein results only in transient myocardial inflammation, but not autoimmunity (90). Models of EAM produced by inoculating rodents with cardiac actin or cardiac c protein require instead of FCA the use of *Klebsiella pneumoniae* O3 lipopolysaccharide (LPS) (91, 92). Neither actin nor c protein will produce EAM by themselves. Substitution of *K. pneumoniae* O3 LPS with LPS derived from other *Klebsiella* species, *Escherichia coli*, and *Salmonella* also fails to produce EAM (91, 92). Timing of adjuvant administration is also critical, as inoculating animals with FCA several days or weeks prior to the myocarditic mixture prevents EAM (93). In other words, every animal model of RHD and AM requires some concurrent combination of antigens, not a single, simple molecular mimic. Such concurrent combinations may be an important clue to the natural etiologies of RHD and AM: the difference between uncomplicated GAS or CX infections and those that go on to induce chronic autoimmune disease might be that the presence of specific co-infections that act as “adjuvants” for GAS or CX (40, 94).

Epidemiological studies of RHD and AM suggest that those people who develop autoimmunity do differ in having co-infections from those people who do not develop autoimmunity, who are characterized by uncomplicated monoinfections. Epidemiological studies demonstrate that 80–100% of RHD cases occurred when CX infection was present concurrently, while uncomplicated GAS infection was almost never associated with development of RHD (95–97). Conversely, 65–80% of AM cases that present with CX infection were complicated with a concurrent GAS infection (32, 98, 99). Moreover, AM patients differ significantly from patients with uncomplicated monoinfections with a variety of viruses and bacteria in producing T cell receptors (TCR) against both CX and GAS (48). In addition, between 12 and 42% cases of AM are associated with multiple, concurrent viral infections (11, 14, 100) often involving CX, parvovirus B19, and HHV6. Possible bacterial co-infections such as GAS were not considered in these viral studies and may also have been present.

### Beyond molecular mimicry to antigenic complementarity

There are several ways in which co-infections may play crucial roles in transforming a simple GAS or CX infection into one that induces autoimmune disease. To begin with, each infection may act as an “adjuvant” for the other, each initiating an immune response that induces localized cytokine release at the site of co-infection. The resulting synergy would produce the so-called “bystander effect” that has been postulated to work in conjunction with MMT to initiate release of alloantigens and to produce autoimmune disease. Such co-infections might also alter the Th1/Th2 balance. It appears that uncomplicated infections tend to produce a Th2 response, whereas a shift toward a Th1 response is more likely to result in autoimmunity [e.g., (29, 101)]. One of the key roles played by adjuvants in animal models of RHD and AM is to shift the immune response toward a Th1 mode (102–104). Co-infections may also produce synergistic effects that can promote autoimmunity (105, 106). In fact, animals co-inoculated with GAS and CX are far more likely to develop severe carditis than are animals inoculated with either pathogen separately (99, 107).

The possibility that GAS and CX act synergistically (and that other co-infections may do so as well) opens up a new possibility that extends MM to antigenic complementarity. Root-Bernstein has demonstrated that GAS induces antibody responses that are complementary to CX antibodies, so that the two sets of idiotypic antibodies act like an idiotype–anti-idiotype pair (40). This antigenic complementarity is further mirrored in the induction of pairs of complementary TCR that mimic both GAS and CX antigens (48). The complementarity of the antigens presented by CX and GAS to the immune system is mirrored in the molecular complementarity of some of the key extracellular host proteins found in this study. GAS mimics laminin, CAR, and myosin while CX mimics collagen IV. CAR and CX are molecularly complementary, CX using CAR as a receptor. GAS and collagen IV are complementary, GAS using collagen IV as a receptor. Laminin and collagen IV are themselves molecularly complementary. Laminin binds specifically to collagen IV to form the fundamental structure of the basement membrane (108–114) and this self-assembly can be interrupted by antibodies against either laminin or collagen IV resulting in failure of the integrity of the membrane (112). Actin (which is an epitope mimic of CX) and myosin (which is an epitope mimic of GAS) are also molecularly complementary; actin and myosin bind to each other to form the actomyosin complex essential to contractile muscle function (115–117). The fact that GAS is antigenically complementary to CX, that laminin is complementary to collagen IV, that myosin is complementary to actin, and that all six are known targets of autoantibodies in RHD/AM, suggests that beyond MM, the role of antigenic complementarity in the pathogenesis of autoimmune forms of carditis must also be considered.

### A new model of RHD/AM pathogenesis

**Figure 10:**
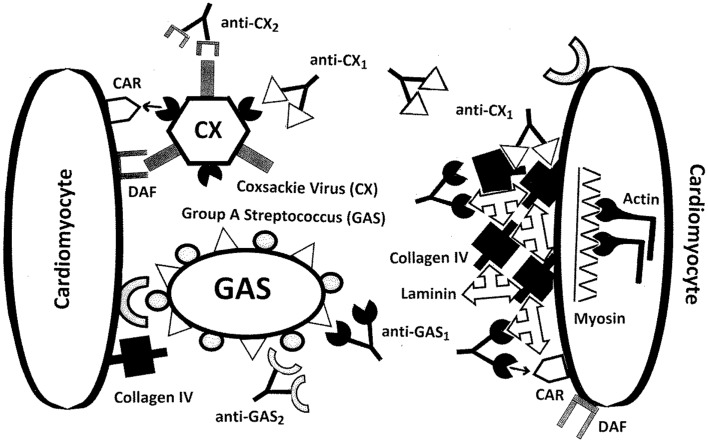
**Illustration of the ways in which molecular mimicry between coxsackie virus antigens (CX) and/or group A streptococci (GAS) antigens and extracellular proteins of cardiomyocytes may result in the production of autoantibodies**. CX uses the coxsackie and adenovirus receptor (CAR) as well as the complement decay-accelerating (DAF or CD55) protein as receptors to target cardiomyocytes. Some CX proteins mimic collagen IV as well as the beta 1 adrenergic receptor (B1AR, not shown), so that anti-CX immune responses may also target collagen IV and B1AR. GAS uses collagen IV and various other cardiac proteins as receptors. The M protein of GAS mimics laminins so that antibodies against GAS may also target cardiac laminins. In addition, GAS M protein and laminins share epitope similarities with CAR, so that CAR may be an additional target of GAS antibodies. Note that although the illustration shows antibodies, the same sets of interactions could occur with T cell receptors (TCR).

**Figure 11:**
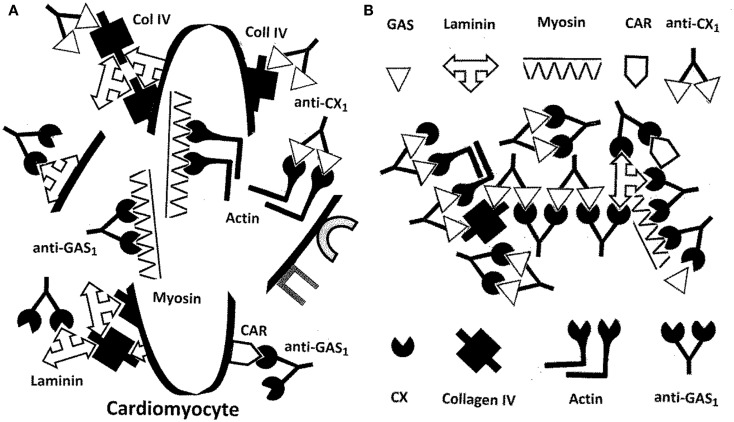
**(A)** Illustration of the destructive effects that antibodies from Figure 10 would have on cardiomyocytes. Autoantibodies against GAS and/or CX would target basement membrane proteins such as laminin and collagen IV and possibly cardiomyocyte receptors such as CAR and B1AR. Cardiomyocyte membrane integrity would be compromised permitting antibodies to move on to target actin, myosin, and other hidden antigens. Because of the molecular mimicry between the GAS M protein, laminins, CAR, and myosin, epitope drift would result in a range of immune responses against all four. Similarities between CX epitopes, collagen IV, B1AR, and actin would lead, through epitope drift, to the similar evolution of the immune response from the pathogen to the cell membrane to hidden antigens inside. Note again, as in Figure 10 that although this process is illustrated with antibodies, the same sets of interactions could be mediated through TCR. **(B)** Antigens on GAS, especially the M protein, mimic laminins, myosin, and the coxsackie and adenovirus receptor (CAR). Antigens on coxsackie viruses (CX) mimic collagen IV and actin as well as the beta 1 adrenergic receptor (B1AR – not shown). In consequence, antibodies against CX also recognize collagen IV and actin (and possibly B1AR, not yet demonstrated), while antibodies against GAS M protein also recognize laminin, myosin, and possibly CAR, not yet demonstrated. It also follows that antibodies against GAS are complementary to antibodies against CX, so that anti-CX mimics GAS, laminin, myosin, and CAR; and antibodies against CX are complementary to antibodies against GAS, so that anti-GAS mimics CX epitopes, collagen IV, actin, and possible B1AR. The antigenic complementarity of GAS for CX will therefore lead their antibodies to precipitate each other and to form circulating immune complexes involving GAS and CX proteins as well as the entire range of host protein targets. The immune system will be unable to distinguish “self” from “non-self” leading to abrogation of tolerance and the induction of autoimmune disease. Note once again that the same consequences would occur for T-cell mediated responses as are illustrated here for antibodies.

The extension of MM to extracellular proteins and the incorporation of antigenic complementarity suggest a new explanation of RHD/AM pathogenesis that is illustrated in Figures 10 and 11. Infections with either GAS or CX localize at cardiomyocytes producing an immune response that cross-reacts with laminin and/or CAR (in the case of GAS) or collagen IV and/or B1AR (in the case of CX) (Figure 10). Damage to the extracellular matrix destabilizes cardiomyocyte structure permitting myosin and actin to be released (Figure 11A). Anti-GAS antibodies and T cells cross-reactive with laminins and/or CAR, in turn, begin to target the more antigenic cardiac myosin. Anti-CX antibodies and T cells cross-reactive with collagen IV and/or B1AR will begin to target the more antigenic actin. Even after GAS and CX infections are eliminated, their antibodies and T cells will continue to be stimulated by the presence of an ongoing supply of both extracellular and intracellular “self” proteins. Thus, the immune response evolves from GAS-CX to laminin-collagen to myosin-actin through epitope drift.

To this point, it is possible to interpret Figures 10 and 11A as if either GAS or CX can independently induce autoimmunity against heart tissue, but such a model does not explain what makes a GAS or CX infection that does not lead to autoimmune disease different from one that does; cannot account for the rarity of autoimmune disease following GAS or CX infections; and cannot account for the need of “adjuvants” (or perhaps some sort of hyperinflammation) in animal modes of RHD and AM. It is proposed that these difficulties can be addressed by assuming that autoimmune disease only follows combined infections of GAS with CX (or perhaps with other combinations of cardiotropic pathogens), as is consistent with both human epidemiology and the animal experiments of Kogut et al. (99) and Pearce (107). Thus, it is possible to re-interpret Figures 10 and 11A as showing what might happen if GAS and CX were present simultaneously. Figure 11B then explores some of the unique consequences of such a combined infection.

If both GAS and CX are present simultaneously, rather than as independent infections, unique consequences ensue (Figure 11B). In the first place, the anti-GAS immune response (whether antibody or T cell) will be antigenically complementary to the anti-CX immune response (antibody or T cell). The resulting antibodies and/or T cells will act like idiotype–anti-idiotype pairs, even though each response has been initiated as an idiotypic one. These complementary immune responses will do several unusual things. First, they will neutralize each other forming immune complexes and stimulating a hyperinflammatory response. This hyperinflammatory response will take place specifically at the cardiomyocyte cell matrix and at the GAS and CX cardiomyocyte receptors (Figure 10). Not only will the immune system be less able to clear the co-infection of GAS-CX due to co-neutralization of the immune responses, but the hyperinflammation will cause excess cardiac damage, further driven by release of hidden antigens such as myosin and actin (Figure 11A). Notably, circulating immune complexes (CIC) will be formed that are composed not only both sets of antibodies but also any of the GAS, CX, and cardiac antigens that may be present (Figure 11B). These CIC will stimulate further damage not only at heart tissue but also in the liver and kidneys.

Most importantly, this new model provides a specific mechanism by which the distinction between “self” and “non-self” can be abrogated. When the immune system having to process two antigenically complementary pathogens, each of which mimic complementary “self” proteins, the result is the production of complementary immune responses (antibody and/or T-cell mediated) each of which mimics host proteins as well as one of the pathogens. The immune system becomes “confused,” tolerance must be abrogated in order to respond to the pathogens, and autoimmune disease follows.

The model is both clinically and experimentally testable. Clinically, it should be found that people who develop RHD/AM differ from those with uncomplicated GAS or CX infections in either having both infections simultaneously, of having some other combination of co-infections that are cardiotropic. Experimentally, it should be possible to induce experimental forms of RHD and AM using a combination of GAS-CX infection, or even by using inactivated GAS with inactivated CX. Passive transfer of experimental RHD/AM should be achievable by infecting one group of rodents with GAS, another with CX and then transferring antibodies and/or T cells from both groups to a single set of recipients. The mono-infected animals should not develop RHD/AM, but the recipients of the combined GAS-CX antibodies and/or T cells should develop autoimmune disease.

## Conclusion

In sum, broadening the view of molecular (epitope) mimicry in RHD/AM beyond myosin and actin suggests a model of pathogenesis that accounts not only for the experiments and observations stemming from MMT but also for the much broader set of data that MMT cannot explain. RHD and AM are rare despite the frequency of GAS and CX infections because each pathogen requires an antigenically complementary co-infection, which is often each other. Combining an extended form of MM with antigenic complementarity also provides a specific mechanism by which tolerance can be broken and the specific set of autoantigens that are subsequently targeted explained, which MMT cannot do by itself.

It is worth noting that the model suggested is predicated on a significant departure from Koch’s postulates. Koch’s postulates have, of course, been the standard for demonstrating disease causation for more than a century but assume that each disease has a single pathogenic cause. The proposition that RHD, AM, and perhaps other autoimmune diseases are due to specific combinations of pathogens runs contrary to the isolation of the single “causative” agent of disease. A multifactorial set of “disease postulates” will be required instead, in which it is demonstrated that more than one pathogen is associated with an autoimmune disease, that no single one of these pathogens can induce the autoimmune disease, but that a combination of the pathogens does induce autoimmune disease (48, 106, 118).

## Conflict of Interest Statement

The author declares that the research was conducted in the absence of any commercial or financial relationships that could be construed as a potential conflict of interest.
